# Nasal resistance and OSA

**DOI:** 10.1016/S1808-8694(15)31036-3

**Published:** 2015-10-19

**Authors:** Levon Mekhitarian Neto

**Affiliations:** a Mestrando (sócio)

## REPLY

I thank you for your interest and comment on my article posted on “Revista Brasileira de Otorrinoralingologia “.

The OSA has a variety of causes, with anatomic abnormalties on the oropharyngeal and hypopharyngeal airways. We believe that these abnormalties are caused by the oral respiration since childhood, causing problems in the tongue posture, abnormal deglutition, and bite alterations, with orthognatic and morphological facial alterations.

In our series of cases the structural alterations of the nasal cavity had high incidence in patients with OSA, although I did not state that this was an OSA cause, but we emphasized that these nasal abnormalties must be studied and corrected, whenever possible, to increase CPAP tolerance.

Sincerly,

Levon Mekhitarian Neto

